# The DDN Catalytic Motif Is Required for Metnase Functions in Non-homologous End Joining (NHEJ) Repair and Replication Restart*

**DOI:** 10.1074/jbc.M113.533216

**Published:** 2014-02-25

**Authors:** Hyun-Suk Kim, Qiujia Chen, Sung-Kyung Kim, Jac A. Nickoloff, Robert Hromas, Millie M. Georgiadis, Suk-Hee Lee

**Affiliations:** From the ‡ Department of Biochemistry and Molecular Biology, Indiana University School of Medicine, Indianapolis, Indiana 46202,; the § Department of Environmental and Radiological Health Sciences, Colorado State University, Fort Collins, Colorado 80523,; the ¶ Department of Medicine, University of Florida and Shands Health Care System, Gainesville, Florida 32610, and; the ‖ Department of Chemistry and Chemical Biology, Indiana University Purdue University Indianapolis, Indianapolis, Indiana 46202

**Keywords:** DNA Binding Protein, DNA Damage, DNA Enzymes, DNA Repair, DNA Replication

## Abstract

Metnase (or SETMAR) arose from a chimeric fusion of the *Hsmar1* transposase downstream of a protein methylase in anthropoid primates. Although the Metnase transposase domain has been largely conserved, its catalytic motif (DDN) differs from the DDD motif of related transposases, which may be important for its role as a DNA repair factor and its enzymatic activities. Here, we show that substitution of DDN^610^ with either DDD^610^ or DDE^610^ significantly reduced *in vivo* functions of Metnase in NHEJ repair and accelerated restart of replication forks. We next tested whether the DDD or DDE mutants cleave single-strand extensions and flaps in partial duplex DNA and pseudo-Tyr structures that mimic stalled replication forks. Neither substrate is cleaved by the DDD or DDE mutant, under the conditions where wild-type Metnase effectively cleaves ssDNA overhangs. We then characterized the ssDNA-binding activity of the Metnase transposase domain and found that the catalytic domain binds ssDNA but not dsDNA, whereas dsDNA binding activity resides in the helix-turn-helix DNA binding domain. Substitution of Asn-610 with either Asp or Glu within the transposase domain significantly reduces ssDNA binding activity. Collectively, our results suggest that a single mutation DDN^610^ → DDD^610^, which restores the ancestral catalytic site, results in loss of function in Metnase.

## Teaser

**Background:** Metnase, a transposase-containing DNA repair protein, retains DNA cleavage activity with a DDN motif.

**Results:** Substitution with the ancestral transposase DDD/DDE catalytic motif results in a decrease in ssDNA binding and ss-overhang cleavage activities.

**Conclusion:** The DDN motif is required for Metnase DNA repair activities.

**Significance:** Understanding the requirements for catalytic activity provides insights on how Metnase functions as a DNA repair protein.

## Introduction

The human genome retains ∼200 copies of the full-length DNA transposon *Hsmar1* (1, 2); most of these have diverged from the consensus largely independently of each other and are now defective (2, 3). A remarkable exception to this loss of functionality was found in a domesticated copy of the *Hsmar1*, where the transposase gene was fused to a SET gene (2, 4, 5). Although domestication of transposable elements is not uncommon (6–8), the events leading to the chimeric SET transposase gene, termed Metnase (also known as *SETMAR*), are unusual in that they occurred late in primate evolution and ultimately resulted in the evolution of a protein with a new DNA repair function (4, 5). Metnase has multiple functional roles including non-homologous end joining (NHEJ)^2^ repair, DNA integration, chromosomal decatenation, and restart of stalled replication forks activities that involve its transposase domain (9–13). It does not, however, retain the ability to function as a transposase (3, 14).

The transposase domain of Metnase was first reported to retain the terminal inverted repeat (TIR)-specific DNA-binding activity but lack DNA cleavage activity (4). This finding was rationalized based on the fact that the highly conserved DDD motif is DDN in Metnase and thus lacks the third acidic residue predicted to be involved in metal binding. The *mariner* family of transposases, from which Metnase evolved, all contain a DDD motif and substitution with DDE results in loss of transposition activity (15). However, later studies reported by two groups independently suggest that Metnase retains DNA cleavage activity (3, 16) despite the loss of the third D within the catalytic motif. Although the Metnase transposase lacks robust 5′ end processing activity catalyzed by Mg^2+^, it was reported to have some activity with Mn^2+^ (3). The DDD Metnase mutant had detectable 5′ end processing activity with Mg^2+^ but much higher activity catalyzed in the presence of Mn^2+^ (3). The major defect in transposase activities found for Metnase was 3′ end processing activity (3). Although the Metnase transposase is 94% identical to the predicted ancestral sequence (2), substitution of the DDN to DDD motif is not sufficient to restore transposase activity (14). Furthermore, full-length Metnase purified from mammalian cells does not appear to retain the same DNA cleavage activity (9, 16) as reported for its transposase domain (3).

**FIGURE 1.**
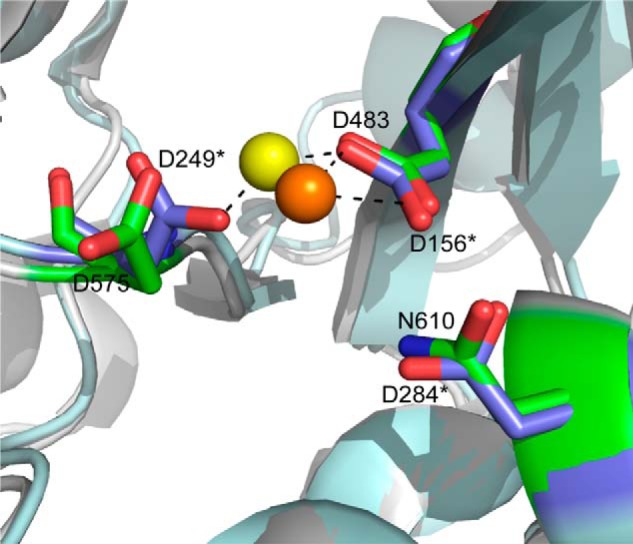
**The active site of the Metnase transposase catalytic domain (Protein Data Bank code 3K9J) is shown superimposed on the same region from Mos1 (Protein Data Bank 2F7T).** Catalytically important residues in Metnase include Asp-483, Asp-575, and Asn-610 shown in a stick model with carbons (*green*), oxygens (*red*), nitrogens (*blue*), and a *gray ribbon* for the surrounding protein structure. In this structure, Ca^2+^ (*orange sphere*) is coordinated by Asp-483. Mos1 residues are indicated with an *asterisk* and include Asp-156, Asp-249, and Asp-284, shown in a stick model with carbon (*blue*) and oxygen (*red*) and the surrounding protein structure shown in a light blue ribbon. Mg^2+^ (*yellow sphere*) is coordinated by Asp-156 and Asp-249 in this structure.

The *mariner* family of transposases was classified as using a two metal ion mechanism involving coordination through the DDD motif (17) based largely on comparison with the Tn5 synaptic complex transposition intermediate structure (18), which contains two Mn^2+^ atoms bound in the active site coordinated by three acidic residues. These enzymes are known to be Mg^2+^-dependent. However, many of the crystal structures reported to date for *mariner*-derived transposases contain one rather than two metal ions bound to the active site (19–21), suggesting that binding of the second metal ion may require the presence of bound substrate. A recent structure reported for the catalytic domain of Mos1 bound to two metal ions and the drug raltegravir is consistent with binding of the second metal in the presence of a bound ligand in the active site (22). By analogy, the proposed model for metal binding suggests that in Metnase Asp-483 would coordinate two metal ions, whereas Asp-575 and Asn-610 (or substituted Asp or Glu) would be involved in coordinating only one of the two metal ions (see Fig. 1 for comparison of Metnase and Mos1 active sites). In the present study, we determined that Asn-610 within the catalytic domain plays an essential role in ssDNA binding activity, which is not only crucial for ss-overhang cleavage but also necessary for function of Metnase in DSB repair and restart of blocked replication forks.

## EXPERIMENTAL PROCEDURES

### 

#### 

##### Cell Lines, RNAi Suppression of Metnase, and Expression of WT and Mutant Metnase

Cell lines were cultured in DMEM with 10% (v/v) fetal bovine serum supplemented with 100 units/ml penicillin and 100 mg/ml streptomycin (Invitrogen). Metnase was overexpressed in HEK293 cells as described (10). FLAG-Metnase overexpression was confirmed by Western blot with a monoclonal antibody (Sigma M1). Metnase was down-regulated by transfecting cells with two siRNAs (5′-GUCGAACAUCAGUUGUGGAAAdTdT-3′, 5′-GCGAUUGACCCUUGAGACUAUdTdT-3′, Dharmacon Research) for 48 h, whereas control cells were transfected with a scrambled siRNA (AllStars negative control siRNA, Qiagen). Metnase expression was measured by RT-PCR and by Western blots using antibodies to native Metnase as described (5).

##### Metnase Purification and Detection by Western Blot

Metnase was purified from HEK293 cells stably expressing FLAG-Metnase as described (10). FLAG-Metnase was detected in cell extracts by Western blotting using a monoclonal antibody (Sigma M1) as described previously (10). Ku80 was used as a loading control and was detected using a monoclonal antibody (Santa Cruz Biotechnology).

##### DNA Cleavage Assay

For preparation of DNA substrates used in DNA cleavage assays, oligonucleotides were mixed and annealed together for 5′-flap DNA (5′-CGATACTGAGCGTCACGGACTCTGCCTCAAGACGGTAGTCAACGTGTTACAGACTTGATG-3′; 5′-GATGTCAAGCAGTCCTAACTTTGAGGCAGAGTCCGTGACGCTCAGTATCG-3′; 5′-CATCAAGTCTGTAACACGTTGACTACCGTC-3′), 5′-fully replicated fork DNA (5′-CGATACTGAGCGTCACGGACTCTGCCTCAAGACGGTAGTCAACGTGTTACAGACTTGATG-3′; 5′-GATGTCAAGCAGTCCTAACTTTGAGGCAGAGTCCGTGACGCTCAGTATCG-3′; 5′-CATCAAGTCTGTAACACGTTGACTACCGTC-3′; 5′-AGTTAGGACTGCTTGACATC-3′), 5-overhang DNA (5′-GCAGTGGCTATCGTATAGTATTAGGTTGGTGACCCCGTAAGGAAAGTTTT-3′; 5′-AAAACTTTCCTTACGGGGTCACCAACCTAATA-3′); 5′-TIR DNA (5′-GCAGTGATTTAGGTTGGTGCAAAAGTAATTGCGGTTTTCGATACTGAGCG-3′; 5′-AAAACCGCAATTACTTTTGCACCAACCTAAATCGCTCAGTATCG-3′); 3′-TIR DNA (5′-ATTTAGGTTGGTGCAAAAGTAATTGCGGTTTTCGATACTGAGCGGCAGTG-3′; 5′-AAAACCGCAATTACTTTTGCACCAACCTAAATCGCTCAGTATCG-3′). DNA cleavage was monitored as described previously (9). Briefly, reaction mixtures (20 μl) containing 50 mm Tris-HCl (pH 7.5), 5 mm DTT, 5% glycerol, BSA (10 μg), and 2 mm MgCl_2_ were incubated with indicated amounts of WT or mutant Metnase in the presence of 200 fmol of 5′-^32^P-labeled DNA. After incubation at 37 °C for indicated amount of time, reaction mixtures were resolved by electrophoresis using 12% polyacrylamide gels containing 8 m urea, and cleavage products were detected by autoradiography.

##### EMSA of Protein-DNA Interaction

Duplex DNA was labeled with [γ-^32^P]ATP (3,000 Ci/mmol) and T4 polynucleotide kinase (Roche Applied Science) according to the manufacturer's instructions. The indicated amount of purified Metnase was incubated with 200 fmol of 5′-^32^P-labeled DNA at room temperature for 15 min in a reaction mixture containing 50 mm HEPES-KOH (pH 7.8), poly(dI:dC) (0.2 μg), bovine serum albumin (0.2 μg/μl), and 50 mm NaCl. The Metnase-DNA complex was analyzed on either a 5% polyacrylamide gel in 0.5× TBE or 1% vertical agarose gel in 1× TBE. The gels were dried, and protein binding was detected by autoradiography.

##### Purification of Metnase Transposase Domains

The Metnase transposase (aa 329–671) and catalytic domain (aa 329–440) were inserted into the pET28b-derived pSUMO vector (23) at BamHI and XhoI sites. These vectors express recombinant protein with an N-terminal His_6_-tagged SUMO fusion partner, which was cleaved using the SUMO-specific protease Ulp1 during the purification to yield the Metnase domain of interest. Metnase, including aa 329–671, was amplified by PCR using the forward primer 5′- ATATCGGATCCATGAAAATGATGTTAGACAAAAAGCAAATTCG and reverse primer 5′-TATAGCTCGAGTTAATCAAAATAGGAACCATTACAATC (Integrated DNA Technologies, Inc., Coralville, Iowa). For Metnase aa 329–440, the forward primer was the same as above, and the reverse primer was 5′-TATAGCTCGAGTTACACCTTTCCAATTTGCTTCAAATGTC. Using WT Metnase aa 329–671 as the template, Metnase aa 329–671 N610D and N610E were generated using the QuikChange II site-directed mutagenesis kit (Agilent Technologies). Proteins were expressed in *Rosetta* cells (Novagen) following induction with 1 mm isopropyl 1-thio-β-d-galactopyranoside at 15 °C overnight. Cells were harvested, resuspended in lysis buffer (50 mm phosphate, pH 7.8, 300 mm NaCl, and 10 mm imidazole), and lysed by using a French press (Aminco) with three passes at 1,000 psi. The crude cell lysates were centrifuged at 35,000 rpm for 30 min at 4 °C, and clear lysates were applied to a nickel-nitrilotriacetic acid column, followed by Ulp1 protease cleavage to liberate Metnase from the fusion protein. The elution fractions were then loaded to an Sp-Sepharose ion exchange column and finally a gel filtration column (Superdex 200 16/60 prep). The C-terminal catalytic domain Metnase (aa 433–671) was expressed and purified as described previously (16). In brief, the protein was purified by nickel-nitrilotriacetic acid chromatography followed by Sp-Sepharose ion exchange chromatography. After thrombin digestion to remove the N-terminal His tag, a second Sp-Sepharose chromatography purification step was performed to obtain the pure protein.

##### Purification of Mos1 Proteins

The original plasmid containing the Mos1 transposase coding sequence was purchased from the Addgene website (plasmid ID 34874) (24). However, this plasmid contains a 51-bp insertion within the Mos1 ORF. To delete this insertion, the fragment was first subcloned into the pET28b-SUMO-tagged vector (23). The insert was then deleted using the QuikChange II site-directed mutagenesis kit (Agilent Technologies). The primers used for PCR amplifying the fragment from the original plasmid were 5′-ATATCGGATCCATGTCGAGTTTCGTGCCGAATAAAGAGC-3′ (BamH I site, *underlined*), 5′-TATAGCTCGAGTTATTCAAAGTATTTGCCGTCGCTCGCG-3′ (XhoI site, *underlined*) (Integrated DNA Technologies, Inc., Coralville, Iowa). The new plasmid was subjected to deletion, using primers 5′-CTCTGCGAGTTGTTTTTGCGTTTGAGCATCGTCTTC-3′ and 5′-GAAGACGATGCTCAAACGCAAAAACAACTCGCAGAG-3′. The final plasmid pET28b-SUMO-tag-Mos1 (WT) was validated by sequencing. To construct the Mos1 (D284N) mutant plasmid, PCR mutagenesis with the primers 5′-CGAATAGGTGGTAATTGGATGGGGCCAGGTC-3′ and 5′-GACCTGGCCCCATCCAATTACCACCTATTCG-3′ was conducted on the basis of Mos1 (WT) plasmid, using the above site-directed mutagenesis kit as well. Mos1 protein overexpression and purification were done using the same procedures described above for Metnase transposase proteins.

##### Fluorescence Polarization Assay

Reaction mixtures (50 μl) containing 50 mm HEPES, pH 7.0, 150 mm NaCl, and 10 nm rhodamine-labeled DNA probe were incubated with varying concentrations of Metnase. TIR DNA sequences were as follows: 5′-(rhodamine) (C6 amino)-AACCGCAATTACTTTTGCACCAACC (Metnase binding site *underlined*) and 5′-TCAGTAGGTTGGTGCAAAAGTAATTGCGGTT were annealed to form a partial duplex DNA with a 5′-6 nt overhang; A 5′-(rhodamine) (C6 amino)-AACCGCAATTACTTTTGCACCAACCTAA-3′ oligonucleotide was annealed to its complementary sequence to make 28-bp blunt-ended dsDNA. Oligonucleotides were purchased from Midland Certified Reagent Company, Inc. (Midland, TX). Measurements were conducted on the Envision^TM^ 2102 Multilabel Plate Reader (PerkinElmer Life Science, Chemical Genomics Core Facility at Indiana University School of Medicine). The data were curve-fitted, and *K_d_* values for the binding activity were calculated using the one site saturation ligand binding equation (SigmaPlot, version 11.2). ssDNA binding activity was assayed using a 25-nt rhodamine-labeled TIR DNA oligonucleotide: 5′-(Rhodamine) (C6-amino)-AACCGCAATTACTTTTGCACCAACC.

##### DNA End-joining Assays

For end-joining assays coupled to *Escherichia coli* colony formation, reactions (100 ml) contained 50 mm Tris-HCl (pH 7.5), 0.5 mm magnesium acetate, 60 mm potassium acetate, 2 mm ATP, 1 mm DTT, and 100 mg/ml BSA. Where indicated, 2.5 mm dNTPs were included in the reaction mixtures. Cell-free extracts were preincubated for 5 min at 37 °C before addition of 1.0 mg of DNA substrate. Following incubation for 1 h at 37 °C, DNA products were deproteinized, purified by QIAprep Kit (Qiagen, Valencia, CA), transformed into *E. coli*, and colonies were scored as described (5). DNA integration was analyzed by the ability of cells to pass foreign non-homologous DNA containing a selectable marker to progeny. pRNA/U6.Hygro plasmid was transfected into HEK293 cells stably transfected with empty pFlag2 vector or WT or mutant Metnase expression vector calcium phosphate transfection as described (10). All experiments were performed three times in triplicate.

##### γ-H2AX Nuclear Foci

Cells (2.0 × 10^5^ cells/well) were grown on poly-d-lysine-coated coverslips (neuVitro, 18-mm diameter) and 48 h later were treated with 2 mm hydroxyurea (HU) for 18 h, washed three times with growth media, and incubated in fresh media for 0, 2, 6, and 24 h. After briefly washing cells with PBS, cells were fixed for 5 min in ice-cold methanol at −20 °C. The slips were then blocked for 30 min with 5% BSA in PBS and incubated with a mouse anti γ-H2AX monoclonal antibody (Millipore, 05-636) for 1.5 h at room temperature. The slides were washed four times with 0.2% Tween 20 and incubated for 1 h at room temperature with a donkey anti-mouse Alexa Fluor 488 secondary antibody (Invitrogen) in a wet chamber covered by aluminum foil. After washing four times with 0.2% Tween 20-PBS, samples were mounted (VECTASHIELD® mounting medium with DAPI, VECTOR Laboratories), and nuclei were visualized using a confocal microscope (Zeiss LSM 510 NLO, 63× oil immersion objective).

##### DNA Fiber Analysis after Dual Labeling of Chromosomal Replication with CldU and IdU

Cells were grown in six-well dishes (6 × 10^5^ per well), and 20 mm IdU was added to growth medium and incubated for 20 min at 37 °C. After washing with fresh medium, cells were treated with 5 mm HU for 60 min or mock-treated. Medium was replaced with fresh medium containing 100 mm CldU, cells were further incubated for indicated times at 37 °C. Cells were harvested, resuspended in PBS, and ∼1000 cells were transferred to a positively charged microscope slide (Superfrost/Plus, Daigger), and processed for DNA fiber analysis as described previously (25). Slides were mounted in PermaFluor aqueous, self-sealing mounting medium (Thermo Scientific), and DNA fibers were visualized using a confocal microscope (Olympus, FV1000D, 63× oil immersion objective). Images were analyzed using the OLYMPUS FLUOVIEW software.

## RESULTS

### 

#### 

##### The DDN^610^ Motif Plays a Critical Role in DSB Repair

**FIGURE 2.**
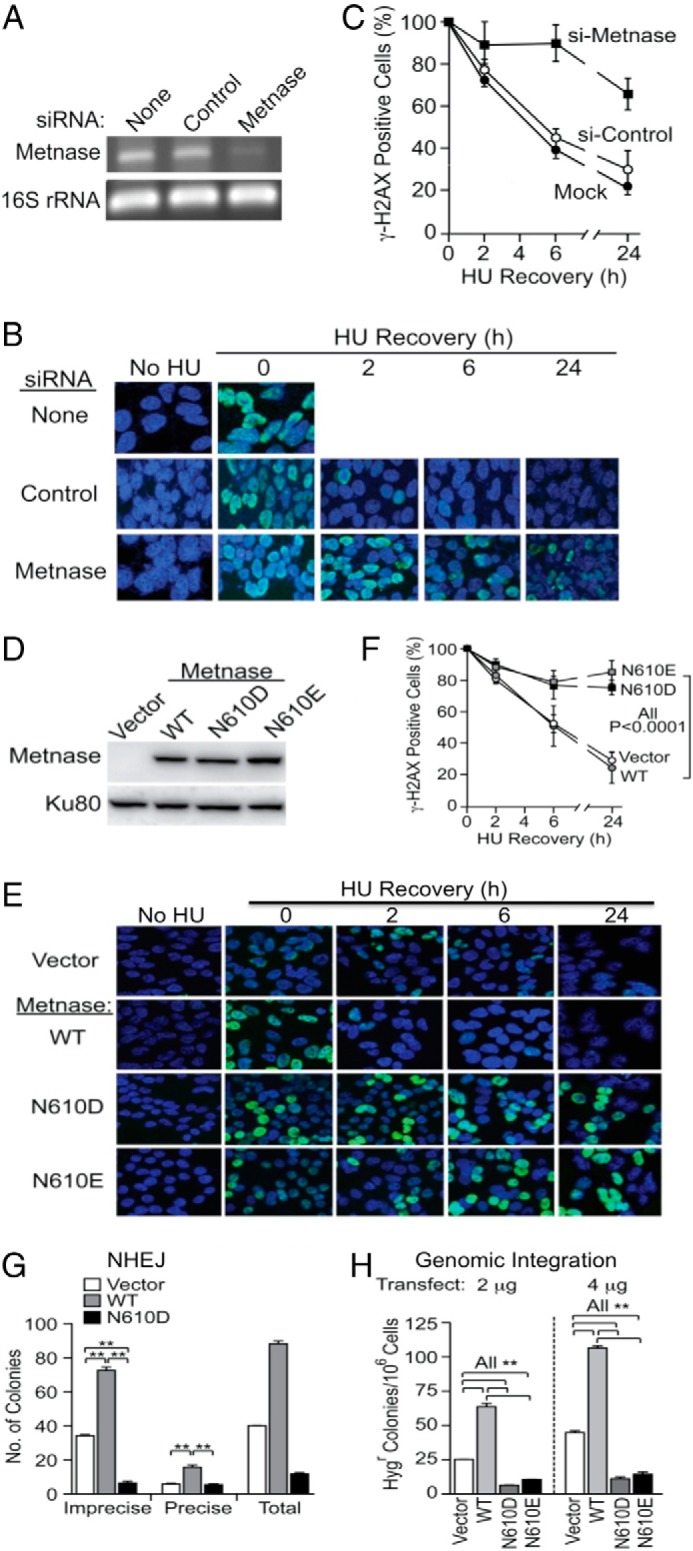
**The catalytic DDN^610^ motif is critical for role of Metnase in DSB repair.** *A*, siRNA knockdown of Metnase analyzed by RT-PCR. *B*, representative images of HEK293 cells mock-transfected (*top*) or transfected with control siRNA (*middle*) or Metnase siRNA (*bottom*). Cells were treated with 2 mm HU for 18 h, released into fresh media at indicated times, stained with DAPI (*blue*) and antibodies to γ-H2AX (*green*), and imaged by confocal microscopy. *C*, percentage of γ-H2AX positive cells treated with either control- or Metnase-specific siRNA. Values are averages ± S.D. for two determinations. *D*, Western blot analysis of FLAG-tagged WT, N610D, and N610E Metnase stably expressed in HEK293 cells using an anti-FLAG monoclonal antibody. Ku80 was used as a loading control. *E*, representative confocal microscope images of HEK293 transfected with pFlagCMV4 vector and HEK293T cells overexpressing WT or mutant Metnase following mock or 18 h treatment with 2 mm HU, released into fresh medium for indicated times, cytospun, stained with DAPI (*blue*) and antibodies to γ-H2AX (*green*), and imaged by confocal microscopy. *F*, quantitation of *E*. Plotted are average percentages (± S.D.) of γ-H2AX-positive cells. An average of >200 cells were counted per slide, six slides per experiment. *G*, *in vitro* DNA end joining in cell-free extracts from HEK293 stably expressing WT or N610D Metnase. Cell extracts were incubated with linearized pBS DNA (1.0 μg), 1 mm MgCl_2_, and 1 mm ATP for 60 min, DNA was isolated and transformed into *E. coli* for colony counts. Values are averages (±S.D.) of three or more DNA end-joining assays. *, *p* < 0.05; **, *p* < 0.01, *t* tests. *H*, the catalytic motif DDN^610^ is crucial for the role of Metnase in DNA end joining coupled to genomic integration. HEK293 cells stably transfected with pFlag2 (vector) or pFlag2 expressing WT, N610D, or N610E Metnase were transfected with 2 or 4 μg) of KpnI-linearized pRNA/U6.Hygro plasmid, Hyg^r^ colonies were scored 14 days later to assess DNA integration. Values are averages (± S.D.) for six determinations. **, *p* < 0.01, *t* tests.

In an effort to understand the functional role of Asn-610, we substituted this position with either Asp or Glu and compared WT Metnase (DDN^610^; Fig. 1) with the DDD^610^ and DDE^610^ mutants in an *in vivo* DSB repair assay by monitoring resolution of phosphorylated H2AX (γ-H2AX), which marks DSBs at collapsed replication forks following 18 h exposure to 2 mm HU (25). Compared with a control-siRNA, HEK293 cells treated with Metnase-siRNA showed 89% reduction in Metnase expression (Fig. 2*A*). As reported previously (25), siRNA knockdown of Metnase caused γ-H2AX signals to persist, and overexpression of WT Metnase caused γ-H2AX signals to disappear more quickly (Fig. 2, *B* and *C*). Thus, WT Metnase promotes repair of DSBs at collapsed replication forks. Interestingly, cells overexpressing N610D or N610E enzymes (Fig. 2*D*) showed persistent γ-H2AX signals for at least 24 h (Fig. 2, *E* and *F*), similar to knockdown cells. This indicates that the WT DDN promotes the resolution of DSB damage, whereas DDD or DDE containing enzymes do not.

We next compared DSB repair activity of WT and mutant Metnase by measuring precise and imprecise NHEJ of transfected plasmid DNA linearized within the β-galactosidase gene (9). As seen previously (5, 9), overexpression of WT Metnase increased precise and total end joining by 2.2- and 2.1-fold, respectively, whereas cells overexpressing Metnase N610D had a dominant negative effect (Fig. 2*G*). Imprecise NHEJ is predominant in this assay, and imprecise NHEJ was 4.4-fold lower with overexpressed Metnase N610D than parent cells, and 9.6-fold lower than cells overexpressing WT Metnase (Fig. 2*G*). Metnase N610D also negatively impacted precise NHEJ, albeit to a lesser degree. Although it is not clear how DNA endonuclease activity contributes to precise end joining, our results indicate that the Metnase DDN^610^ catalytic motif is required for both precise and imprecise plasmid NHEJ.

Metnase also enhances plasmid and viral DNA integration (5, 26), so we next compared integration efficiency in HEK-293 cells overexpressing WT or N610D mutant Metnase. Consistent with previous results (5), stable overexpression of WT Metnase increased genomic integration of plasmid DNA by >2.7 fold. In contrast, overexpression of Metnase N610D again acted as a dominant negative, reducing integration by 2- to 3-fold (Fig. 2*H*). Together, these results indicate that the Asn-610 residue in the Metnase catalytic motif is essential for its *in vivo* roles in NHEJ and DNA integration, whereas mutants with conserved transposase catalytic motifs (DDD or DDE) inhibited these processes.

##### The DDN^610^ Motif Is Critical for Accelerating the Restart of Stalled Replication Forks

**FIGURE 3.**
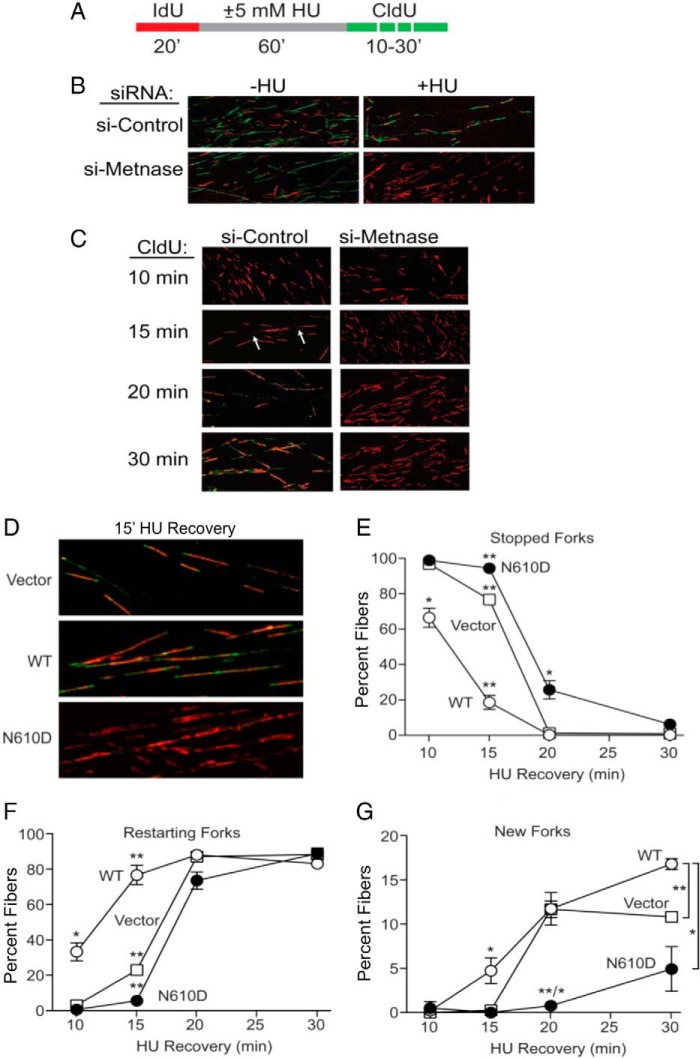
**The Metnase catalytic DDN^610^ motif has a critical role in replication fork restart following HU treatment.** *A*, dual labeling protocol for DNA fiber analysis. Cells were pulse-labeled with IdU (*red*) for 20 min (*20*′), treated with 0 or 5 mm HU for 60 min, and labeled with CldU (*green*) for the indicated times. *B*, representative confocal microscope images of replication tracks from HEK293 cells treated with either control- (*top*) or Metnase-specific siRNA (*bottom*). Cells were treated or mock treated with HU and pulse-labeled with CldU for 15 min. *C*, as in *B* but CldU pulse labeled for 10, 15, 20, or 30 min. *Arrows* indicate short green patches representing early fork restart. *D*, representative images of replication tracks from HEK293 cells stably transfected with empty vector control (*top*) or with WT or N610D mutant Metnase vector (*middle* and *bottom*) and pulse-labeled with CldU for 15 min following 60 min HU treatment. *E–G*, average percentages (± S.D.) of stopped (*red* only), restarted (*red* plus *green*), and new forks (*green* only) for three independent determinations. *, *p* < 0.05; **, *p* < 0.01, *t* tests.

Metnase promotes restart of replication forks stalled by treatment with HU (25), although its precise role is not clear. To further characterize Metnase function in fork restart, we carried out a kinetic analysis using DNA fiber assays with cells transfected with either control or Metnase siRNA. Cells were incubated with IdU for 20 min prior to treatment with 5 mm HU for 1 h and then released into fresh media in the presence of CldU and harvested at different times to determine the percentages of forks that failed to restart (stopped forks), restarted forks, and new forks initiated post HU treatment (Fig. 3*A*). In the absence of HU, control and Metnase knockdown cells showed similar patterns of IdU and CldU incorporated into DNA fibers, but only control cells incorporated CldU after HU (Fig. 3*B*), as reported previously (25). Kinetic analysis revealed that control cells restarted forks within 15 min of HU release (Fig. 3*C*, see *arrows*), whereas Metnase-knockdown cells showed no fork restart within 30 min of HU release (Fig. 3*C*).

We used this same procedure to compare fork restart kinetics in parent HEK-293 cells and derivatives expressing WT or N610D mutant Metnase (Fig. 3*D*). As with Metnase knockdown (Fig. 3*C*), the N610D mutant showed no detectable restart of stalled replication forks 15 min after HU release (Fig. 3*D*). Further kinetic analysis revealed that overexpression of WT Metnase accelerated fork restart, but overexpression of the N610D mutant significantly delayed fork restart (Fig. 3, *E* and *F*). Interestingly, 30 min after HU release, the N610D mutant fully recovered, showing high percentages of restarted forks similar to the control (parental) cells and cells expressing WT Metnase (Fig. 3, *E* and *F*). This result indicates that the Metnase DDN motif does not affect overall restart efficiency, but it does accelerate fork restart. In addition, cells expressing N610D mutant Metnase showed a significantly reduced capacity to initiate new forks after release from HU (Fig. 3*G*). Together, these results indicate that the Metnase DDN motif has critical roles in promoting rapid fork restart and new origin firing upon release from replication stress. Metnase associates with PCNA, the RAD9 stress response factor (25), and Chk1 (27), and these factors may regulate Metnase functions in restarting stalled forks after brief HU exposure (Fig. 3), in late responses to resolve collapsed forks after prolonged HU exposure (Fig. 2, *B* and *E*), and in initiating new replication forks during recovery from replication stress (Fig. 3*G*).

##### DDN^610^ Is Critical for ss-overhang Cleavage Activity but Not End Cleavage Activity in Partial Duplex DNA

**FIGURE 4.**
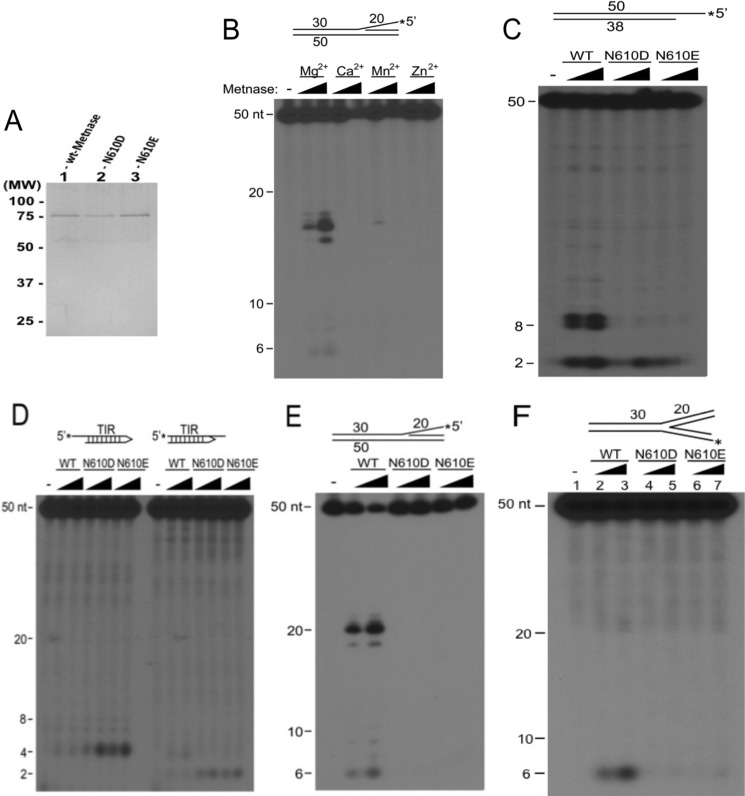
**Metnase cleavage of ss-overhangs requires Mg^2+^ and the DDN^610^ motif.** *A*, silver staining of purified WT and mutant Metnase (50 ng) following 10% SDS-PAGE. *B*, ss-overhang cleavage activity of Metnase in the presence of different metal ion. Reaction mixtures (20 μl) containing 50 fmol of 5′-^32^P-labeled 5′-flap DNA and 100 ng of WT-Metnase in the presence of 2 and 5 mm of indicated metal ion were incubated for 60 min at 37 °C, and cleavage products were analyzed by 12% PAGE containing 8 m urea. DNA size makers are indicated on the *left. C*, *E*, and *F*, DNA cleavage of 5′-overhang partial duplex DNA (*C*), 5′-flap DNA (*E*), and a fully replicated fork DNA (*F*) by WT Metnase, N610D, or N610E mutant Metnase (50 and 100 ng). *D*, Metnase DDD and DDE mutants show enhanced end cleavage activity with TIR DNA with either 3′- or 5′-ss-overhang. Reaction mixtures (20 μl) containing 50 fmol of 5′-^32^P-labeled TIR DNA were incubated with 50 and 100 ng of either WT or mutant Metnase in the presence of 2 mm MgCl_2_. After incubation for 60 min at 37 °C, cleavage products were analyzed by 12% PAGE containing 8 m urea. DNA sizes are indicated on the *left*.

Although the Metnase catalytic domain is very similar to related transposases (19), Metnase has unique ss-overhang cleavage activity (9). To further clarify the role of the Metnase DDN^610^ catalytic motif in DNA repair and replication fork restart, we purified WT and catalytic motif mutant Metnase (Fig. 4*A*) and compared *in vitro* cleavage activities on a 5′-flap substrate that mimics an arrested replication fork, and partial duplex DNA with a 5′-ssDNA extension. First, unlike eukaryotic transposases (21, 28, 29) in which Mn^2+^ can substitute for Mg^2+^ as the catalytic metal ion, only Mg^2+^ supported Metnase-mediated 5′-flap cleavage activity (Fig. 4*B*). We then compared cleavage of 5′-ssDNA extensions in partial duplex DNA by WT Metnase and mutant DDD^610^ or DDE^610^ enzymes. Unlike WT Metnase, neither DDD nor DDE mutant cleaved near the ssDNA-dsDNA junction, but both retained ssDNA end cleavage activity, removing two nt from the 5′-end (Fig. 4*C*). Metnase retains several *mariner* transposase functions, including TIR binding and TIR 5′-end cleavage (3, 14). Interestingly, N610D and N610E mutant enzymes showed enhanced 5′-TIR end cleavage activity relative to WT Metnase (Fig. 4*D*). We also compared WT and DDD/DDE mutant Metnase cleavage of the 5′-flap DNA substrate. WT Metnase efficiently removes this ssDNA flap, but no activity was observed with N610D or N610E mutants (Fig. 4*E*). We also carried out DNA cleavage of WT Metnase and the mutants with a fully replicated fork DNA (Fig. 4*F*). The result was somewhat similar to DNA cleavage activity with flap DNA (Fig. 4*E*), except that no branch cleavage was observed with a fully replicated DNA. Together, these results indicate that substitution of Asn-610 with Asp or Glu restores end cleavage activity linked to transposase function but destroys 5′-flap cleavage activity associated with DNA repair and replication fork restart.

##### Asn-610 Plays a Crucial Role in Metnase-ssDNA Binding

**FIGURE 5.**
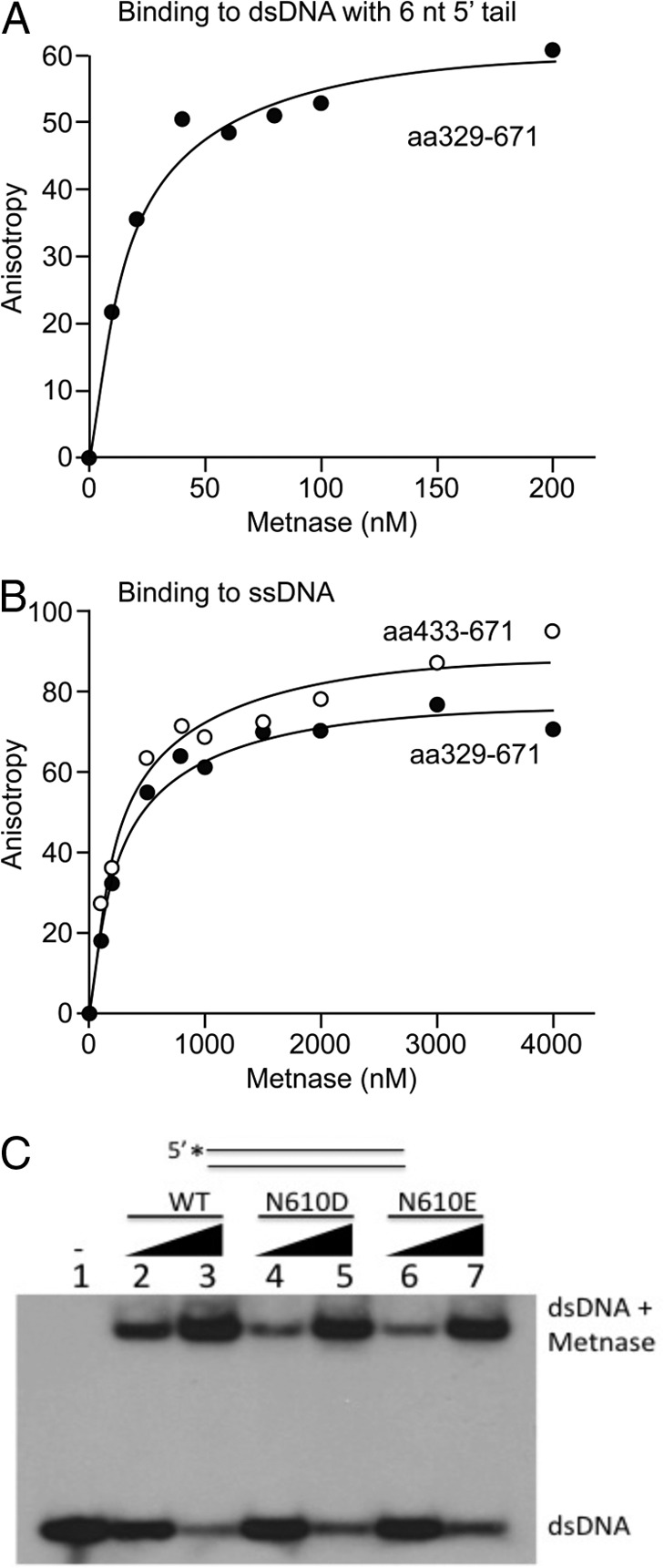
**Binding curves of Metnase fragments to different DNA probes as measured by fluorescence anisotropy.** *A*, Metnase transposase domain (aa 329–671) with 5′ 6-nt dsDNA (25/31-mer), *K_d_* of 11.9 ± 2.5 nm. *B*, Metnase transposase domain (aa 329–671) and the catalytic domain (aa 433–671) with ssDNA (25 nt), *K_d_* of 277 ± 26 nm and 295 ± 47 nm, respectively. The equilibrium binding data were analyzed by nonlinear curve fitting using the one site saturation ligand binding equation (SigmaPlot, version 11.2). The *K_d_* values are presented as *K_d_* ± S.E. S.E. represents the error calculated for the determination of the *K_d_* value based on the fit of the data. At least two independent assays were done for each sample, and duplicate measurements were averaged for each concentration of protein used. *C*, WT-Metnase and DDD/DDE mutant bind TIR DNA. Gel mobility shift analysis of WT and mutant Metnase (0.05 or 0.1 μg) incubated with 5′-^32^P-labeled TIR DNA (200 fmol) resolved on a 0.8% agarose gel.

Transposases include two functional domains: two N-terminal helix-turn-helix motifs responsible for TIR binding and a C-terminal catalytic domain that mediates cleavage and joining reactions during transposition (30). Although Metnase retains TIR binding activity, its catalytic properties are distinct from ancestral transposases (Fig. 4) (9, 14, 16). To further understand Metnase-mediated 5′-flap cleavage, we determined binding affinities of the Metnase transposase domain (aa 329–671) to different DNA substrates using fluorescence polarization-based equilibrium DNA binding assays (16). It was not possible to perform fluorescence polarization assays using the full-length Metnase due to the large amounts of protein required to perform these experiments. Because the DNA-binding activity resides within the transposase domain, purified transposase domain was used for these measurements. The Metnase transposase domain binds dsDNA and a partial duplex DNA with 6-nt extension with high affinity (*K_d_*, 7.9 and 11.9 nm, respectively; Fig. 5*A* and Table 1). Surprisingly, the Metnase transposase domain also binds ssDNA with relatively high affinity (*K_d_* = 277 nm; Fig. 5*B*). Given that helix-turn-helix motifs bind dsDNA, the catalytic domain might directly interact with ssDNA to effect cleavage of 5′-ssDNA extensions in partial duplex DNA, and 5′-flaps in pseudo-Tyr structures. We therefore determined binding affinities of the DNA binding (aa 329–440) and catalytic (aa 433–671) domains for ss- and dsDNA. The catalytic domain bound ssDNA with similar affinity (*K_d_* of 295 nm) to the full-length transposase (Fig. 5*B*, Table 1), but the DNA binding domain had no detectable ssDNA binding activity (Table 1). However, the DNA-binding domain bound dsDNA with high affinity (*K_d_*, 3.2 nm), whereas the catalytic domain showed at least 1,500-fold lower dsDNA binding activity (Table 1). Together, these results suggest that the catalytic domain mediates Metnase binding to ssDNA, whereas the helix-turn-helix domains are responsible for binding to dsDNA. The idea that Metnase binds dsDNA through its helix-turn-helix DNA binding domain, and not the DDN catalytic domain, is supported by the observation that DDD and DDE mutant Metnase binding to dsDNA in an electrophoretic mobility shift assay is indistinguishable from WT DDN Metnase (Fig. 5*C*).

**TABLE 1:** **Summary of *K_d_* values of Metnase fragments with ssDNA (25 nt) and blunt-ended dsDNA (28/28-mer) measured by fluorescence anisotropy** The dissociation constant (*K_d_*) was calculated by non-linear regression and plotted (SigmaPlot, version 11.2). The *K_d_* values are presented as *K_d_* ±S.E. Duplicate measurements were made for each protein concentration used.

Oligonucleotide	Transposase domain (WT)	DNA binding domain (WT)	Catalytic domain (WT)	Transposase domain (N610D)	Transposase domain (N610E)
ssDNA	277 ± 26 nm	No binding	295 ± 47 nm	3.3 ± 1.1 μm	3.2 ± 0.4 μm
Blunt-ended dsDNA	7.9 ± 1.9 nm	3.2 ± 0.9 nm	4.7 ± 0.8 μm	ND*^a^*	ND

ND, not determined.

Having established the roles for the DNA binding and catalytic domains in binding ds- and ssDNA, respectively, we next determined the effects of N610D, N610E, and N610A substitutions on ssDNA binding activity of the Metnase transposase domain. Both N610D and N610E mutations significantly increased *K_d_* values (>3 μm; Table 1). Thus, whereas WT Metnase binds ssDNA with moderately high affinity, the DDD and DDE mutants possess significantly reduced ssDNA binding activity, suggesting that the catalytic pocket, and Asn-610 in particular, plays a crucial role in ssDNA binding. We also sought to characterize the ssDNA-binding activity of a related *mariner* transposase, Mos1, and the effect of substituting Asn for Asp-284, the residue equivalent to Asn-610 in Metnase. Fluorescence polarization assays were done as described for Metnase. Surprisingly, both Mos1 and Mos1 D284N enzymes bound ssDNA with similar high affinities (*K_d_*, 33 ± 9 nm and 33 ± 12 nm, respectively).

## DISCUSSION

Metnase is a multifunctional protein with DNA endonuclease, histone methyltransferase, and interaction with DNA ligase IV, TopoIIα, Pso4, and NBS1, all of which likely contribute to its role in promoting NHEJ, DNA integration, TopoIIα-dependent chromosome decatenation, and restart of blocked replication fork (5, 9, 10, 13, 16, 19, 25–27, 31–35). Metnase knockdown confers sensitivity to ionizing radiation as well as anticancer drugs causing DSB damage (5, 13), whereas human cells stably transfected with Metnase-shRNA vectors either cease to proliferate after 8–12 weeks or revert to normal (5, 25), indicating that Metnase promotes proliferation of human cells. Because Metnase interacts with TopoIIα and regulates its activity, cells lacking Metnase show a defect in decatenation checkpoint (12).

The results described here provide new insights on the role of a negatively charged residue in the catalytic site of a Mg^2+^-dependent nuclease. By analogy to the Tn5 transposase (19), one might predict that Asp-610 in ancestral Metnase coordinated a second metal ion. It was therefore somewhat unexpected that substitution of Asp or Glu for Asn-610 negatively impacts the functional activities of Metnase. It is possible that the neutral Asn residue at a non-Mg^2+^ coordinating position in the catalytic motif allows a stable interaction with negatively charged ssDNA extensions and flaps, which in turn, is necessary for ssDNA cleavage. In this model, replacing the neutral Asn-610 residue with negative charges in the N610D and N610E mutants abrogates ssDNA binding and cleavage of ssDNA flaps and extensions but enhances cleavage near the ssDNA ends required for transposase function. These results suggest that Asn-610 within the active site dictates substrate specificity. As Mg^2+^ strongly prefers coordination by carboxylate ligands, it is extremely unlikely that the functional role of Asn-610 in the DNA cleavage activities observed for Metnase is to coordinate Mg^2+^. In addition, Metnase cleavage activities are not supported by Mn^2+^ for which Asn-610 could serve as a coordinating ligand.

Having established that WT Metnase containing Asn-610 supports while the Asp-610 enzyme decreases functionally relevant activities, it was of interest to investigate the ssDNA-binding properties of a related mariner transposase, Mos1. Both WT and D284N Mos1 bind ssDNA with high affinity, ∼10-fold more tightly than WT Metnase. Potentially, these results suggest that the binding site for ssDNA in Mos1 is not the same as that in Metnase and may not involve residue 284 directly in Mos1. It is also tempting to speculate that very high affinity for ssDNA is required for 3′-end processing in transposases. This idea is consistent with the loss of 3′-end processing in Metnase and acquisition of 5′-ss flap cleavage activity upon substitution of Asn for Asp at residue 610, restoring moderate binding affinity for ssDNA.

The Metnase SET domain is responsible for methylation of histone H3 at lysines 4 and 36, which are associated with “open” chromatin and may increase accessibility of repair factors to damaged DNA (5). However, a lack of DNA endonuclease activity directly affects DNA end processing that significantly lowers end-joining activity (9). Considering that N610D/N610E mutants lacking ss-overhang cleavage activity failed to support the role of Metnase in the restart of replication forks (Figs. 3 and 4), Metnase-mediated endonuclease activity is likely involved in fork restart activity, although it is possible that other activities of Metnase such as protein-protein interactions were affected in the DDD or DDE mutants *in vivo*. Our findings support the hypothesis that the DDN catalytic motif is essential for the role of Metnase in accelerating restart of stalled replication forks and promoting the repair of DSBs at collapsed replication forks, both of which contribute to genome stability in unstressed cells (*i.e.* at fragile sites) and in cells experiencing replication stress from genotoxic chemicals and radiation.

## Footnotes

2 The abbreviations used are:

NHEJ: non-homologous end joining
DDN/DDD/DDE: catalytic motifs
DSB: double strand break
dsDNA: double strand DNA
HU: hydroxyurea
ssDNA: single strand DNA
TIR: terminal inverted repeat
aa: amino acids
nt: nucleotide(s).
